# Professor Henry Reginald Noel

**Published:** 1878-02

**Authors:** 


### EDITORIAL, ETC.

---

### OBITUARY.

*Professor Henry Reginald Noel.*—Few clearer and brighter minds have ever graced the medical profession of Baltimore than that of PROF. HENRY REGINALD NOEL, who died at his residence, in Virginia, on the 26th day of January.

After graduating, with distinguished honors, at the University of Virginia, Dr. Noel came to reside in Baltimore, in 1858, where he quickly won for himself, in an unusual measure, the respect and admiration of the profession and the public.

At the beginning of the late war, his sympathies led him to espouse the cause of his native State, Virginia, and he at once abandoned a growing practice to accept a Surgeon's commission in the Confederate Army, where he served with marked ability, —in the Brigade of General Henry A. Wise, and other important positions,—to the close of the war. Shortly after the close of the war, Dr. Noel returned to Baltimore, and resumed the practice of medicine.

In 1865 he was elected to the Professorship of Physiology in the Baltimore College of Dental Surgery, where he soon made for himself a high reputation as a lecturer and *teacher*. He also filled the chair of Physiology in the College of Physicians and Surgeons of Baltimore for two years. Early in the Spring of 1876, his health had become so greatly impaired, that he was compelled to relinquish both his professional duties and his private practice,—retiring to his farm in Virginia, where he continued to reside until his death, last month.

As a teacher and lecturer Dr. Noel was eminently successful. His lectures were not only prepared with a most laborious and conscientious care, but were delivered with such earnest and genuine enthusiasm, as to make them doubly effective. He was gifted with the ability to express himself with a clearness and definiteness not often witnessed; and he always succeeded in inspiring his classes with an enthusiasm kindred to his own. We feel sure, there are not a few, dentists and physicians, throughout the country, who will acknowledge to a debt of deep gratitude to the teachings and example of Professor Noel.

As a member of various local medical Societies, the Medical and Chirurgical Faculty of Maryland, and the American Medical Association, and as a frequent contributor to medical and dental periodical literature, Dr. Noel was widely known to the two professions as an indefatigable worker, a profound and clear thinker, and an enthusiastic student in the science of medicine. He was specially characterized by the originality and independence of his opinions, and the vigor and earnestness with which he defended them. Although he had been suffering for the past fifteen years, from the inroads of pulmonary consumption, —to which he finally succumbed,—his ambitious nature and active energy, were superior to all bodily infirmity; and, in the intervals of a busy and exacting practice, he found time to keep himself well informed as to the discoveries and advances, not only of medical but of various cognate sciences.

To the brilliant qualities of intellect with which Dr. Noel was endowed, were added a kind, genial disposition; a frank manliness; a freedom from all little meannesses, and a brave constancy in friendships, that made him not only admired, but beloved by his colleagues, his classes, his patients, and indeed, by all with whom he was brought into contact. E. L. H.

At a meeting of the Faculty of the Baltimore College of Dental Surgery, called to give expression to their feeling at the death of Dr. Henry R. Noel, late Professor of Physiology in that School, it was—

*Resolved,* That in the early death of Prof. Noel the College and the members of the Faculty have lost a cordial friend; one whose warm heart and enthusiastic nature endeared him to the affectionate regard of all who knew him, no less heartily than his knowledge, wisdom and high gifts as a teacher commended him to all whom he taught.

*Resolved,* That in respect to his memory the usual badge of mourning shall be worn by the faculty for thirty days.

*Resolved,* That we tender to his family our most heartfelt sympathy in this their hour of affliction.

